# Enhancing Quality of Life in Ostomized Patients Through Smart-Glasses-Supported Health Education: A Pre-Post Study

**DOI:** 10.3390/healthcare14020216

**Published:** 2026-01-15

**Authors:** Emilio Rubén Pego Pérez, Tomás Mendoza Caamaño, David Rey-Bretal, Noelia Gerbaudo-González, Nuria Martínez Laranga, Manuel Gandoy Crego, Raquel Rodríguez-González

**Affiliations:** 1 Faculty of Nursing, University of Santiago de Compostela, Avda. Xoán XXIII s/n, 15703 Santiago de Compostela, Spain; tomas.mendoza.caamano@sergas.es (T.M.C.); davidrey.bretal@usc.es (D.R.-B.); noelia.gerbaudo@usc.es (N.G.-G.); manuel.gandoy@usc.es (M.G.C.); raquel.rodriguez@usc.es (R.R.-G.); 2 Research Group of Dependence, Gerontology and Geriatrics, Faculty of Nursing, University of Santiago de Compostela, Avda. Xoán XXIII s/n, 15703 Santiago de Compostela, Spain; 3 Nursing Consultation for Ostomies, Galician Health Service, University Hospital Complex of Santiago de Compostela, 15703 Santiago de Compostela, Spain; nuria.martinez.laranga@sergas.es

**Keywords:** ostomy, health education, smart glasses, self-care, quality of life, nursing interventions

## Abstract

**Background**: Ostomy care consultations are essential for promoting patient autonomy and quality-of-life. The integration of innovative technologies may enhance health education and support effective self-care among ostomized patients. Objective: To evaluate the impact of a nursing-led health education intervention supported by smart-glasses on the quality of life of ostomized patients. **Methods**: A pre–post quasi-experimental design was employed with 14 patients who had undergone digestive surgery resulting in an ostomy. The intervention consisted of a single 60-min session comprising three phases: (1) assessment of baseline knowledge on ostomy management, (2) personalized feedback, and (3) a hands-on workshop using Vuzix© smart-glasses to demonstrate ostomy care techniques. Quality of life was assessed using the SF-36 questionnaire before and after the intervention. **Results**: The intervention significantly improved overall SF-36 scores, with notable advancements in emotional role (78.57 ± 36.06 to 97.44 ± 9.25, d = 10.54), mental health (79.14 ± 20.10 to 87.38 ± 13.94, d = 6.27), and vitality (69.29 ± 20.56 to 71.15 ± 16.98, d = 4.19). Social function remained high throughout the study, while bodily pain showed a slight decline. A strong correlation (ρ = 0.923, *p* = 0.001) was observed between pre- and post-intervention quality of life scores. **Conclusions**: The findings suggest that integrating smart-glasses into nursing-led health education may enhance the quality of life and self-care capabilities of ostomized patients. However, the small sample size, lack of a control group, and exploratory nature of the study limit the generalizability of the results. Further research is needed to validate these findings in larger, controlled trials.

## 1. Introduction

Ostomy care is a critical aspect of healthcare for patients who undergo digestive surgeries, as it directly impacts their quality of life and ability to achieve autonomy in self-care. Effective ostomy management requires patients to acquire specific technical skills, such as stoma cleaning, pouch application, and complication detection, which can be challenging and anxiety inducing, particularly in the absence of adequate education and support. The Nursing Best Practice Guidelines highlight the importance of early, tailored health education during ostomy care consultations to foster patient independence and improve quality of life [1]. Despite this, access to high-quality ostomy care remains limited in many settings, leading to disparities in patient outcomes and increased healthcare costs [2,3].

Patients with ostomies often face significant physical, emotional, and psychological challenges. These challenges include managing the physical discomfort associated with stoma care, coping with changes in body image, and addressing the social stigma that can arise from living with an ostomy [4]. Studies have shown that inadequate education and support can lead to poor self-care practices, heightened anxiety, and reduced quality of life [5,6,7]. For instance, a recent study by Ketterer et al. [2] demonstrated that individuals living with a stoma in rural areas reported significantly lower quality of life due to limited access to specialized care and educational resources. Traditional educational methods, while effective to some extent, may not fully address the diverse needs of patients, particularly those who struggle with understanding complex care instructions or lack access to specialized services [8,9]. Furthermore, these methods often fail to provide the hands-on, interactive learning experiences necessary for mastering ostomy care techniques and building patient confidence [10].

From an epidemiological standpoint, the increasing prevalence of chronic conditions such as inflammatory bowel disease, coupled with longer life expectancy and expanded cancer screening programs, has resulted in a growing number of patients requiring invasive surgeries and ostomy creation [4].

Innovative technologies, such as smart-glasses, have emerged as promising tools to enhance health education. These wearable devices offer immersive, first-person perspectives, enabling patients to observe and replicate self-care techniques from their own point of view. This approach has demonstrated benefits in improving skill acquisition, reducing anxiety, and increasing confidence in performing technical tasks [11,12]. For example, Muroi et al. [13] demonstrated that 85% of students who used smart-glass-recorded videos for learning technical procedures showed significant improvements in their understanding and performance of the tasks. Similarly, Aranda-García et al. [6] reported that smart-glasses enhanced the accuracy of technical skills during cardiopulmonary resuscitation simulations, achieving a 90% effective chest compression rate compared to 68% in the control group. These findings under-score the potential of smart-glasses to improve health education outcomes across di-verse clinical contexts, including ostomy care.

In addition to their technical benefits, smart-glasses may address the educational challenges faced by ostomized patients in complex environments, such as public settings or during non-routine care situations. These environments often exacerbate patient anxiety and hinder learning, making the interactive and immersive nature of smart-glasses particularly valuable [5]. Aspiotis et al. [7] found that immersive technologies reduce stress in complex scenarios. Araújo et al. [8] and Kim et al. [9] reported high levels of satisfaction, which were associated with improved adherence to self-care practices. Similarly, Sato et al. [14] observed enhanced adherence among medical students, highlighting the potential of these interventions across diverse populations. In this context, adherence refers to patients’ ability to consistently perform self-care tasks, a crucial factor in achieving optimal health outcomes.

Emotional and psychological support also play a vital role in improving the quality of life for ostomized patients. Krouse et al. [15] highlighted that comprehensive ostomy self-management programs can significantly improve patients’ emotional well-being and their ability to adapt to life with an ostomy. Araújo et al. [8] also emphasized that wearable electronic devices, such as smart-glasses, can increase motivation and confidence by providing real-time feedback and enhancing learning experiences. These technologies can also reduce stress in complex scenarios, as demonstrated by Aspiotis et al. [7], who found that immersive technologies can effectively alleviate anxiety in high-stress environments.

However, the application of smart-glasses in ostomy care education remains underexplored, despite their potential benefits for improving patient outcomes. Ostomized patients frequently experience anxiety when learning new care techniques. Immersive technologies, such as smart-glasses, provide real-time, visually engaging training materials that can enhance learning and reduce stress by offering patients a first-person perspective during demonstrations [15]. Moreover, the use of smart-glasses may help bridge the gap in access to specialized ostomy care services, particularly in underserved areas, by enabling remote education and support [16].

This pilot study aims to address the critical need for effective and accessible health education interventions for ostomized patients by evaluating the impact of a nursing-led health education program supported by smart-glasses. By targeting both the technical and psychological challenges of ostomy care, this study seeks to enhance patients’ quality of life and self-care capabilities while contributing to the growing body of evidence on the use of immersive technologies in healthcare.

## 2. Materials and Methods

### 2.1. Study Design, Population and Sample

This study was conducted as an exploratory, quasi-experimental, pre–post pilot study aimed at evaluating the potential impact of a nursing-led health education intervention supported by smart-glasses on the quality of life of ostomized patients. The exploratory nature of the study reflects its primary goal of evaluating the feasibility and preliminary effects of the intervention, without aiming to establish definitive conclusions or causal relationships. The absence of a control group and the small sample size further emphasize the pilot and proof-of-concept nature of this research.

The study was conducted in the Santiago de Compostela and Barbanza Health Area, with participants recruited from the University Hospital Complex of Santiago de Compostela (CHUS) between December 2024 to May 2025. The inclusion criteria were: (1) adult patients (≥18 years old), (2) individuals who had undergone digestive surgery resulting in a permanent or temporary ostomy for more than six months, (3) regular attendees of the ostomy consultation, and (4) those who provided informed consent. Patients with terminal illness or cognitive impairment were excluded from the study.

A total of 22 patients were initially recruited for the study using convenience sampling. However, 14 participants met the inclusion criteria and completed both pre- and post-intervention assessments. All participants were recruited from the same hospital to ensure consistency in clinical and educational practices. The sample size was not calculated a priori due to the pilot nature of the study. However, the effect size was retrospectively calculated to assess the magnitude of the intervention’s impact. The sociodemographic characteristics of the participants, such as age, sex, marital status, educational level, and employment status, were collected using a structured data collection form. Clinical data related to the ostomy, including the type of surgery, ostomy duration, and ostomy age, were also recorded.

### 2.2. Variables

Independent variables included sociodemographic data (age, sex, gender, marital status, educational level, and employment status) and clinical data related to the ostomy (type of surgery, ostomy duration, and ostomy age). Dependent variables were assessed using the Short Form Health Survey (SF-36), which measures quality of life across eight domains: physical function, physical role, bodily pain, general health, vitality, social function, emotional role, and mental health, as well as a health transition item. The validated Spanish version of the SF-36 was used [10]. The questionnaire was administered through face-to-face interviews conducted by trained nursing staff before and after the intervention.

### 2.3. Outcome Measures

The primary outcome of this study was quality of life, measured using the SF-36. Although this tool does not directly evaluate constructs such as self-confidence, autonomy, or self-efficacy, its domains of mental health, social functioning, and emotional role have been widely recognized as indirect indicators of these constructs [11,12]. These dimensions are particularly relevant for assessing the impact of educational interventions on the overall well-being of patients with chronic conditions, including those with ostomies [13,14]. A score of ≥75 points on the SF-36 was considered indicative of a significant improvement in quality of life.

### 2.4. Intervention

The intervention consisted of a single 60-min session, divided into three phases:

Assessment of patient knowledge: Patients’ baseline knowledge of ostomy management was evaluated using a structured questionnaire. The questionnaire included questions on diet, device handling, and stoma complication detection, with responses categorized into low, moderate, or high levels of knowledge.

Personalized feedback: Based on the initial assessment, patients received tailored feedback to address their specific needs and reinforce their understanding of ostomy care. This phase emphasized building confidence and addressing misconceptions.

Hands-on workshop using smart-glasses: Patients participated in a practical session using Vuzix© smart-glasses (Vuzix Corporation©, New York, NY, USA) to enhance their learning experience. The glasses pro-vided a first-person perspective of ostomy care techniques, including bag removal, stoma cleaning, pouch application, and pouch emptying. Four instructional videos were recorded and made available via a digital platform for further review (Video S1).

### 2.5. Data Collect

Data collection was conducted confidentially through structured interviews and patient records during regular ostomy consultations. The STROBE checklist and flow diagram were used to describe the sample. To increase the credibility of the study, the STROBE checklist and the flowchart used to describe the sample have been included (Table S1 and Figure S1).

### 2.6. Ethical Considerations

The inclusion period spanned from the date of approval by the Santiago-Lugo Ethics-Research Committee (registration code 2024/389) and the granting of the Bio-medical Research Study Contract by IDIS until 1 May 2025. The study adhered to Good Clinical Practice Guidelines, the Declaration of Helsinki, the Oviedo Convention, and current data protection regulations. Anonymity and confidentiality of patient data were strictly maintained throughout the study.

### 2.7. Data Analysis

A descriptive analysis was performed using measures of central tendency (mean, M) and dispersion (standard deviation, SD) for quantitative variables, and absolute frequencies and percentages for qualitative variables. Spearman’s correlation and Chi-square tests were used. Statistical analysis was conducted using PASW Statistics (version 23.0; SPSS Inc., Chicago, IL, USA), with a significance level set at *p* < 0.05 (two-tailed).

## 3. Results

A total of 14 patients participated in the study, with a mean age of 57 ± 12.6 years. Half of the participants were male, and the majority were married (78.6%) and had primary or vocational education (78.6%). Clinically, 64.3% of participants had permanent ostomies, and 50% had lived with an ostomy for over two years. Most surgeries were scheduled (57.1%), and 35.7% of the participants had temporary ostomies lasting more than six months (Table 1).

At baseline, participants reported moderate limitations in physical function and physical role, with general health and vitality indicating fair-to-good perceptions. Mental health was adequate in 57.1% of participants. Following the intervention, the mean SF-36 score increased by 5.87 points, from 70.86 ± 21.30 (baseline) to 76.73 ± 12.04. This result represents an 8.3% improvement in overall quality of life scores. Notably, 53.8% of participants reported a very good quality of life after the intervention com-pared to 50% at baseline (Table 1).

Improvements were observed across multiple SF-36 domains. For example, the “Physical Function” domain increased from 72.14 ± 27.65 to 79.62 ± 25.04, a gain of 7.48 points (10.4% increase). Emotional role improved from 78.57 ± 36.06 to 97.44 ± 9.25, representing a gain of 18.87 points or a 24.0% increase, with 100% of participants re-porting no limitations post-intervention compared to 85.7% at baseline. Mental health scores also improved from 79.14 ± 20.10 to 87.38 ± 13.94, a gain of 8.24 points or a 10.4% increase. Additionally, 76.9% of participants reported optimal scores after the intervention compared to 57.1% at baseline.

In the “Vitality” domain, scores increased slightly from 69.29 ± 20.56 to 71.15 ± 16.98, a gain of 1.86 points or a 2.7% improvement. Social function remained high throughout the study, improving slightly from 83.68 ± 19.15 to 84.04 ± 21.45, with a gain of 1.36 points (1.6% increase). In the “Bodily Pain” domain, a slight decline was observed, with scores decreasing from 66.96 ± 23.72 to 64.42 ± 26.14, a reduction of 2.54 points or 3.8% (File S1).

**Figure 1:**
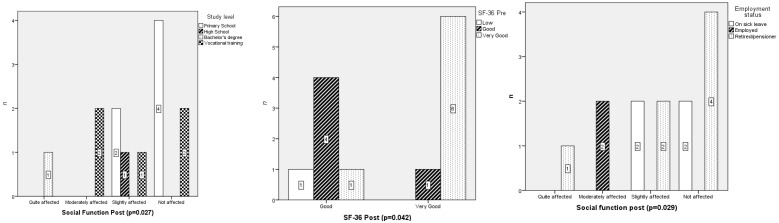
Chi-square test between study level, employment status and social function at second interview; and between Quality of Life (SF-36) pre and post.

The results suggest a strong potential impact of the intervention but warrant further investigation in larger, controlled trials to confirm these findings (Figure 1).

Statistical analyses demonstrated significant improvements in quality of life across several SF-36 domains. The largest effect sizes were observed in the emotional role (d = 10.54), mental health (d = 6.27), and vitality (d = 4.19) domains, as presented in Table 2. However, these large effect sizes should be interpreted cautiously given the small sample size and the uncontrolled design of this pilot study.

There was a strong direct correlation between baseline and post-intervention quality of life scores (ρ = 0.923, *p* = 0.001). For example, the “Physical Function” domain showed a substantial effect size (d = 3.18), with scores increasing by 7.48 points (10.4%). “General Health” improved by 3.74 points (6.4%, d = 2.66), while “Health Transition” increased by 10.17 points (17.8%, d = 2.61). In contrast, “Bodily Pain” decreased by 2.54 points (d = 2.46). All t values were statistically significant (*p* < 0.001), underscoring the robustness of the findings and their clinical relevance (Table 2).

Although the calculated effect sizes for several domains were large, these results should be interpreted with caution. The small sample size, lack of a control group, and pilot nature of the study limit the ability to draw definitive conclusions about the magnitude of the intervention’s impact. These findings should be viewed as preliminary and require further validation in larger, randomized controlled trials.

## 4. Discussion

This pre-post study evaluated health education using smart-glasses as a nursing intervention, suggesting a positive impact on the quality of life of ostomized patients. The findings demonstrate meaningful improvements in several domains of quality of life, particularly in emotional role, mental health, and physical function, highlighting the potential of smart-glasses as an innovative tool in nursing care.

Recent research indicates the effectiveness of smart-glasses in enhancing technical skills and user confidence. In the context of ostomy care, smart-glasses offer immersive educational experiences that may enhance self-care adherence and reduce patient anxiety, as shown in this study [6].

Smart-glasses seem to enhance learning and self-care adherence. Araújo et al. [8] reported increased motivation and confidence, applicable to ostomy care education. Immersive technologies, like virtual reality, have shown potential in reducing stress in high-complexity scenarios, offering potential in ostomy education [7,11].

Similarly, smart-glasses have been shown to improve technical accuracy during complex procedures, such as cardiopulmonary resuscitation, which underscores their potential applicability in supporting ostomy management [6,12,13]. Muroi et al. [13] observed that 85% of students using smart-glass-recorded videos improved their understanding of technical procedures, suggesting this approach might enhance ostomized patients’ ability to learn and practice care techniques effectively [14].

The emotional and educational support provided through this intervention appears to be a key factor in the observed improvements. Krouse et al. [15] observed bet-ter follow-up levels in patients who received such interventions, with Stoma-QOL scores rising from 6.3 ± 1.8 to 7.1 ± 1.7 (*n* = 25). However, Krouse et al. [16] later noted more limited results in telematic interventions, showing no significant differences in overall quality of life except for the physical domain (6.52 ± 1.74; *p* < 0.05; *n* = 54).

This aligns with the findings of Coca et al. [17], who emphasized the role of tailored health education in fostering better adaptation and improved quality of life for ostomized patients. While the improvements in SF-36 scores observed in this study are consistent with prior research, the exploratory nature of this pilot study necessitates cautious interpretation. The lack of a control group and small sample size limit the generalizability of the findings and preclude causal inferences. Other studies have re-ported improvements in SF-36 dimensions, showing a positive impact on patients’ quality of life, aligning with this study [18,19].

Social function scores remained relatively high throughout the study (82.68 ± 19.15 to 84.04 ± 21.45), suggesting activation of the social role dimension and minimal disruption in patients’ social interactions. This highlights the importance of incorporating strategies that promote social connectivity into educational interventions for ostomized patients. The physical role domain showed marginal improvement (73.21 ± 42.14 to 76.92 ± 36.03), contrasting with more pronounced changes observed in other studies [18]. This suggests that while the intervention had a positive impact, additional strategies may be required to achieve greater improvements in physical domains. These results highlight the need for extended follow-up periods to capture long-term effects on physical functionality.

The errors referred to in this study are related primarily to the potential misinterpretation or improper application of ostomy care techniques by patients. These include incorrect stoma cleaning, improper pouch application, and failure to recognize complications such as irritation or leakage. Such errors may arise due to inadequate education, anxiety, or lack of confidence during the learning process. The use of smart-glasses in this intervention aimed to address these challenges by providing patients with immersive, first-person instructional videos that facilitated better under-standing and execution of self-care tasks.

In the bodily pain dimension, this study observed initial values of 66.96 ± 23.72, which worsened slightly to 64.42 ± 26.14 points in the second interview. This decline contrasts with findings from Indrebø et al. [18], who reported progressive relief in pain scores over time. Such discrepancies may reflect differences in intervention duration or sample characteristics.

While most dimensions appeared to show consistent improvement, bodily pain exhibited a slight decline (66.96 ± 23.72 to 64.42 ± 26.14), contrasting with findings by Indrebø et al. [18] who reported progressive relief in pain scores over time. This discrepancy may reflect variations in intervention duration or sample characteristics. The general health domain showed moderate improvement (58.57 ± 23.16 to 62.31 ± 23.42), partially aligning with Wang et al. [19], who observed significant increases in satisfaction within this dimension. Similarly, vitality scores improved slightly (69.29 ± 20.56 to 71.15 ± 16.98), suggesting that more targeted interventions could further enhance energy levels and motivation.

In the vitality dimension, this study observed initial values of 69.29 ± 20.56 im-proving to 71.15 ± 16.98 in the follow-up. Wang et al. [19] reported greater improvement, starting with a lower baseline (37.04 ± 5.28) and achieving higher post-intervention values (75.08 ± 4.86).

In the social function dimension, this study reported higher initial values (82.68 ± 19.15), which improved to 84.04 ± 21.45 points in the second interview. These results reflect transitions from moderate to slightly or not affected categories, suggesting activation of the social role dimension within the framework of quality of life.

Building upon the findings of this study, the observed improvements in quality of life across SF-36 dimensions further support the potential effectiveness of health education interventions using smart-glasses for ostomized people. Specifically, the emotional role dimension demonstrated the most significant enhancement, with scores in-creasing from 78.57 ± 36.06 to 97.44 ± 9.25 points. Similarly, substantial improvements were noted in the mental health domain, with values rising from 79.14 ± 20.10 to 87.38 ± 13.94 points post-intervention. These findings align with prior research that underscores the role of immersive technologies in promoting emotional and psycho-logical well-being, particularly in complex care settings [7,11,20].

In the mental health dimension, this study observed an improvement from initial values of 79.14 ± 20.10 (*n* = 14) to 87.38 ± 13.94 points post-intervention (*n* = 13).

Anxiety is common among ostomized patients, particularly when learning new tasks. Hosseini et al. [11] demonstrated virtual reality’s effectiveness in reducing anxiety before surgical procedures (*p* < 0.01). The ability of smart-glasses to provide immersive, first-person instructional videos may contribute to reducing stress during the learning process, while simultaneously improving technical skills [7,20,21,22].

Despite their potential, smart-glasses are not without limitations. Challenges such as short battery life, connectivity issues, device weight, and user adaptation to advanced technologies must be addressed to ensure successful implementation in clinical practice [9,23,24,25]. Additionally, patients unfamiliar with advanced technologies may require orientation and practice sessions to maximize benefits. Addressing these challenges is essential for successful implementation in health education for ostomized patients [8,12,13,26].

Overall, the findings emphasize the preliminary clinical relevance of smart-glasses as a tool for enhancing self-care adherence, emotional resilience, and technical skills among ostomized patients. However, given the exploratory design and small sample size, these results should be interpreted as preliminary evidence that re-quires further validation in larger, controlled studies. Future research should explore the integration of immersive technologies into personalized educational interventions to address specific challenges in bodily pain and physical functionality, while assessing their long-term impact on health-related quality of life [27].

### Limitations

This study has several limitations: (1) the quasi-experimental design without a control group, constrained by ethical restrictions, limited the ability to establish causal relationships; (2) the brief follow-up period, small sample size, and participant heterogeneity may have introduced biases, affecting the generalizability of the results; (3) external contextual variables and specific intervention components were not analyzed; and (4) the lack of objective measures and a cost-effectiveness analysis reduced the clinical applicability of the findings; (5) while this design allows for the exploration of the intervention’s effects, establishing causality and conducting group comparisons would require controlled designs, such as randomized controlled trials or stepped-wedge models.

## 5. Conclusions

The use of smart-glasses in health education as a nursing intervention in ostomy consultations improved the quality of life of ostomized patients after digestive surgery. The influence of factors such as educational level and employment status on social functioning was confirmed. These results highlighted the effectiveness of educational interventions and nursing care in addressing the physical, emotional, social, and psychological needs of ostomized patients, reinforcing their applicability in clinical practice.

## Figures and Tables

**Table 1:** Central and dispersion measures.

	*n*	%	M	SD	Q1	Q2	Q3	IL	SL
Age	14		57	12.61	48	57.5	67	32	80
Sex									
Man	7	50							
Woman	7	50							
Marital status									
Single	1	7.1							
Married	11	78.6							
Divorces	2	14.3							
Educational level									
Primary education	6	42.9							
Secondary education	1	7.1							
University or higher education	2	14.3							
Vocational training	5	35.7							
Employment status									
Employed	2	14.3							
On sick leave	5	35.7							
Retired/pensioner	7	50							
Type of surgery									
Scheduled	8	57.1							
Emergency	6	42.9							
Duration of ostomy									
Temporary (superior a 6 meses)	5	35.7							
Permanent	9	64.3							
Ostomy age									
<1 year	2	14.3							
1–2 years	5	35.7							
>2 years	7	50							
Quality of Life—1st Interview			70.86	21.30	59.67	75.56	87.51	27	98
Low quality of life	2	14.3							
Good quality of life	5	35.7							
Very good quality of life	7	50							
Quality of Life—2nd Interview			76.73	12.04	70.18	76.61	85.08	55	97
Good quality of life	6	46.2							
Very good quality of life	7	53.8							

M: mean; SD: standard deviation; Q1: first quartile; Q2: second quartile; Q3: third quartile; IL: inferior limit; SL: superior limit.

**Table 2:** t-student results.

Domain	Baseline Mean ± SD	Post-Intervention Mean ± SD	Mean Difference	95% CI (Lower, Upper)	t	df	Sig. (Two-Tailed)	Effect Size (d)
Quality of Life	70.86 ± 21.30	76.73 ± 12.04	5.87	(69.46–84.01)	22.975	12	0.001	6.37
Physical Function	72.14 ± 27.65	79.615 ± 25.04	7.48	(64–94.75)	11.465	12	0.001	3.18
Physical Role	73.21 ± 42.14	76.92 ± 36.029	3.71	(55.15–98.70)	7.698	12	0.001	2.14
Bodily Pain	66.96 ± 23.72	64.423 ± 26.1437	−2.54	(48.625–80.222)	8.885	12	0.001	2.46
General Health	58.57 ± 23.16	62.31 ± 23.418	3.74	(48.16–76.46)	9.593	12	0.001	2.66
Vitality	69.29 ± 20.56	71.15 ± 16.975	1.86	(60.90–81.41)	15.114	12	0.001	4.19
Social Function	83.68 ± 19.15	84.038 ± 21.4461	1.358	(71.079–96.998)	14.129	12	0.001	3.92
Emotional Role	78.57 ± 36.06	97.44 ± 9.245	18.87	(91.85–103.02)	38	12	0.001	10.54
Mental Health	79.14 ± 20.1	87.38 ± 13.938	8.24	(78.96–95.81)	22.606	12	0.001	6.27
Health Transition	57.14 ± 28.47	67.31 ± 25.789	10.17	(51.72–82.89)	9.410	12	0.001	2.61

## Data Availability

The datasets generated and/or analyzed during the current study are not publicly available but can be obtained from the corresponding author upon request, subject to privacy and ethical restrictions.

## Supplementary Materials

The following supporting information can be downloaded at: https://www.mdpi.com/article/10.3390/healthcare14020216/s1, Figure S1: STROBE Flow chart for observational studies; Table S1: STROBE Statement—Checklist of items that should be included in reports of cohort studies; Video S1: Health Education in Ostomies Videos, File S1: (A) Quality-of-life dimensions at first interview. (B) Quality-of-life dimensions at second interview.

## References

[1] Álvarez González D., Crespo Fontán B., Fabeiro Mouriño M.J., García Sánchez R.M., Louzao Méndez S., Pardo Roda P., Parga Lago A., del Carmen Pazos Orosa M., Riveiro García M., Sampayo García B. (2016). Guía de Enfermería Para el Cuidado del Paciente Ostomizado.

[2] Ketterer S.N., Leach M.J., Fraser C. (2021). Factors Associated with Quality of Life Among People Living with a Stoma in Nonmetropolitan Areas. Nurs. Res..

[3] Artola Etxeberría M., García Manzanares M.E., García Moreno V., Martín Fernández M. (2023). Guía de Recomendaciones Prácticas. Ostomía en Atención Primaria.

[4] Cobos Serrano J.L. (2018). Libro Blanco de la Ostomía en España.

[5] Nizum N., Jacob G., Bamford M., Gittens G., White V., Wang D., Naik S., Grinspun D., Ferguson K., Costantini L. (2025). Supporting Adults Who Anticipate or Live with an Ostomy.

[6] Aranda-García S., Barrio-Cortes J., Fernández-Méndez F., Otero-Agra M., Darné M., Herrera-Pedroviejo E., Barcala-Furelos R., Rodríguez-Núñez A. (2023). Dispatcher-assisted BLS for lay bystanders: A pilot study comparing video streaming via smart glasses and telephone instructions. Am. J. Emerg. Med..

[7] Aspiotis V., Miltiadous A., Kalafatakis K., Tzimourta K.D., Giannakeas N., Tsipouras M.G., Peschos D., Glavas E., Tzallas A.T. (2022). Assessing electroencephalography as a stress indicator: A VR high-altitude scenario monitored through EEG and ECG. Sensors.

[8] Araújo A.C., Gardim L., Salma J., Stephen T., Dos Santos S.S., Silva Í.R., de Godoy S., Mendes I.A.C. (2024). Advancing Nursing Education through Wearable Electronic Devices: A Scoping. Nurse Educ. Pract..

[9] Kim S.K., Lee Y., Yoon H., Choi J. (2021). Adaptation of extended reality smart glasses for core nursing skill training among undergraduate nursing students: Usability and feasibility study. J. Med. Internet Res..

[10] Molina-Barea R., Slim M., Calandre E.P. (2024). Health-Related Quality of Life and Psychosocial Variables in Women with Colorectal Pelvic Floor Dysfunction: A Cross-Sectional Study. Healthcare.

[11] Hosseini T., Hooshmandja M., Noaparast M., Mojtahedzadeh R., Mohammadi A. (2024). Virtual reality exposure therapy to decrease anxiety before surgical invasive procedures in hemodialysis patients: An interventional study. BMC Nephrol..

[12] Barcala-Furelos R., Aranda-García S., Otero-Agra M., Fernández-Méndez F., Alonso-Calvete A., Martínez-Isasi S., Greif R., Rodríguez-Núñez A. (2023). Are smart glasses feasible for dispatch prehospital assistance during on-boat cardiac arrest? A pilot simulation study with fishermen. Intern. Emerg. Med..

[13] Muroi K., Kyogoku S., Sakano Y., Sakamoto H., Nakazeko K., Koyama K., Fukunaga I., Hori K., Kotake K., Nojiri S. (2024). An analysis of the effectiveness of reflective learning through watching videos recorded with smart glasses—With multiple views (student, patient, and overall) in radiography education. PLoS ONE.

[14] Sato T., Sandars J., Brown J., Rogers S.N. (2020). Usefulness of smart glasses and point of view for suturing skills training in medical students: Pilot study. BMJ Simul. Technol. Enhanc. Learn..

[15] Krouse R.S., Grant M., McCorkle R., Wendel C.S., Cobb M.D., Tallman N.J., Ercolano E., Sun V., Hibbard J.H., Hornbrook M.C. (2016). A chronic care ostomy self-management program for cancer survivors. Psychooncology.

[16] Krouse R.S., Zhang S., Wendel C.S., Sun V., Grant M., Ercolano E., Hornbrook M.C., Cidav Z., Nehemiah A., Rock M. (2024). A randomized prospective trial of an ostomy telehealth intervention for cancer survivors. Cancer.

[17] Coca C., Fernández de Larrinoa I., Serrano R., García-Llana H. (2015). The impact of specialty practice nursing care on health-related quality of life in persons with ostomies. J. Wound Ostomy Cont. Nurs..

[18] Indrebø K.L., Aasprang A., Olsen T.E., Andersen J.R. (2023). Experiences and results from using a novel clinical feedback system in routine stoma care nurse follow-up of patients with an ostomy: A longitudinal study. J. Patient Rep. Outcomes.

[19] Wang S., Tian H., Xue R. (2021). Using psychological interventions in the nursing care of rectal cancer patients. Am. J. Transl. Res..

[20] Balsam P., Borodzicz S., Malesa K., Puchta D., Tymińska A., Ozierański K., Kołtowski Ł., Peller M., Grabowski M., Filipiak K.J. (2019). OCULUS study: Virtual reality-based education in daily clinical practice. Cardiol. J..

[21] Southworth M.K., Silva J.R., Silva J.N.A. (2020). Use of extended realities in cardiology. Trends Cardiovasc. Med..

[22] Reece R., Bornioli A., Bray I., Newbutt N., Satenstein D., Alford C. (2022). Exposure to green, blue and historic environments and mental well-being: A comparison between virtual reality head-mounted display and flat screen exposure. Int. J. Environ. Res. Public Health.

[23] Baashar Y., Alkawsi G., Wan Ahmad W.N., Alomari M.A., Alhussian H., Tiong S.K. (2023). Towards wearable augmented reality in healthcare: A comparative survey and analysis of head-mounted displays. Int. J. Environ. Res. Public Health.

[24] Fang J.R., Pahwa R., Lyons K.E., Zanotto T., Sosnoff J.J. (2024). Examining the validity of smart glasses in measuring spatiotemporal parameters of gait among people with Parkinson’s disease. Gait Posture.

[25] Marschollek M., Barthel C., Behrends M., Schmeer R., Meyenburg-Altwarg I., Becker M. (2016). Smart Glasses in Nursing Training–Redundant Gadget or Precious Tool? A Pilot Study. Nursing Informatics.

[26] Sekiguchi A., Cao R., Umemori S., Noritake K., Sunaga M., Kinoshita A., Tonami K.I., Nitta H. (2024). Educational effectiveness of remote training with smart glasses for impression-taking. J. Dent. Educ..

[27] Capilla Díaz C., Moya Muñoz N., Matas Terrón J.M., Pérez Morente M.A., Álvarez Serrano M.A., Montoya Juárez R., Hueso-Montoro C. (2022). Evaluation of interventions in people with digestive stoma through the Nursing Interventions Classification. Int. J. Nurs. Knowl..

