# Supplementary material for: Enhancing Quality of Life in Ostomized Patients Through Smart-Glasses-Supported Health Education: A Pre-Post Study
Source: Healthcare (Basel). 2026 Jan 15;14(2):216. doi: 10.3390/healthcare14020216 (PMC12840625; doi:10.3390/healthcare14020216)
Supplement: Supplementary file 1 [file healthcare-14-00216-s001.zip › File S1. (A) Quality-of-life dimensions at first interview. (B) Quality-of-life dimensions at second interview..pdf]

File S1. (A) Quality-of-life dimensions at first interview. (B) Quality-of-life dimensions at second interview.

(A) Quality-of-life dimensions at first interview									
	**n**	**%**	**M**	**SD**	**Q1**	**Q2**	**Q3**	**IL**	**SL**
**Physical Function**			72.14	27.65	51.25	87.50	90	20	100
Very limited	3	21.4							
Slightly limited	10	71.5							
Not limited	1	7.1							
**Physical Role**			73.21	42.14	37.50	100	100	0	100
Limited	3	21.4							
Not limited	11	78.6							
**Bodily Pain**			66.96	23.72	51.25	68.75	90	23	100
Extreme	1	7.1							
Severe	2	14.3							
Moderate	6	42.9							
Mild	4	28.6							
None	1	7.1							
**General Health**			58.57	23.16	35	60	76.25	25	100
Excellent	1	7.1							
Very good	4	28.6							
Good	4	28.6							
Fair	5	35.7							
**Vitality**			69.29	20.56	53.75	70	90	35	100
Low	1	7.1							
Moderate	4	28.6							
Good	5	35.7							
High	2	14.3							
Very high	2	14.3							
**Social Function**			82.68	19.15	57.50	87.50	100	55	100
Moderately affected	5	35.7							
Slightly affected	3	21.4							

Not affected	6	42.9							
**Emotional Role**			78.57	36.06	66.67	100	100	0	100
Affected	2	14.3							
Not affected	12	85.7							
**Mental Health**			79.14	20.10	60	82	100	40	100
Moderately affected	2	14.29							
Moderate	4	28.57							
Adequate	4	28.57							
Optimal	4	28.57							
**Health Transition**			57.14	28.47	50	50	81.25	0	100
Much worse	1	7.14							
Worse	1	7.14							
No change	8	57.15							
Better	1	7.14							
Much better	3	21.43							

(B) Quality-of-life areas at 2nd interview									
	**n**	**%**	**M**	**SD**	**Q1**	**Q2**	**Q3**	**IL**	**SL**
**Physical Function**			79.62	25.04	60	95	97.50	20	100
Very limited	1	7.7							
Slightly limited	9	69.2							
Not limited	3	23.1							
**Physical Role**			76.92	36.03	50	100	100	0	100
Limited	3	23.1							
Not limited	10	76.9							
**Bodily Pain**			64.42	26.14	38.75	70	80	22.50	100
Extreme	2	15.4							
Severe	2	15.4							
Moderate	4	30.8							
Mild	3	23.0							
None	2	15.4							
**General Health**			62.31	23.42	50	65	77.50	10	100
Excellent	1	7.7							
Very good	2	15.4							
Good	8	61.5							
Fair	1	7.7							
Poor	1	7.7							
**Vitality**			71.15	16.98	57.50	70	82.50	45	100
Moderate	3	23.1							
Good	7	53.8							
High	1	7.7							
Very high	2	15.4							
**Social Function**			84.04	21.45	67.50	90	100	37.5	100
Quite affected	1	7.7							
Moderately affected	2	15.4							
Slightly affected	4	30.8							
Not affected	6	46.1							
**Emotional Role**			97.44	9.25	100	100	100	67	100

Not affected	13	100							
**Mental Health**			87.38	13.94	76	92	100	60	100
Moderate	3	23.1							
Adequate	5	38.45							
Optimal	5	38.45							
**Health Transition**			67.31	25.79	50	50	100	25	100
Worse	1	7.7							
No change	6	46.1							
Better	2	15.4							
Much better	4	30.8							
