# Supplementary material for: Enhancing Quality of Life in Ostomized Patients Through Smart-Glasses-Supported Health Education: A Pre-Post Study
Source: Healthcare (Basel). 2026 Jan 15;14(2):216. doi: 10.3390/healthcare14020216 (PMC12840625; doi:10.3390/healthcare14020216)
Supplement: Supplementary file 1 [file healthcare-14-00216-s001.zip › Video S1. Health Education in Ostomies Videos.pdf]

TÍTULO OBRA	AUTOR	URL
Health Education in Ostomies	Mendoza Caamaño, Tomás & Pego Pérez, Emilio Rubén	https://nubeusc-my.sharepoint.com/personal/tomas_mendoza_rai_usc_es/_layouts/15/onedrive.aspx?id=%2Fpersonal%2Ftomas%5Fmendoza%5Frai%5Fusc%5Fes%2FDocuments%2FV%C3%ADdeos%2FEducaci%C3%B3n%20Sanitaria%20en%20Ostom%C3%ADas&viewid=de87c0ef%2D59f2%2D4639%2D9cae%2Dc92dd6a1d80b&ga=1
Video 1. Review of the material for the change of our system	Mendoza Caamaño, Tomás & Pego Pérez, Emilio Rubén	https://nubeusc-my.sharepoint.com/personal/tomas_mendoza_rai_usc_es/_layouts/15/stream.aspx?id=%2Fpersonal%2Ftomas%5Fmendoza%5Frai%5Fusc%5Fes%2FDocuments%2FV%C3%ADdeos%2FEducaci%C3%B3n%20Sanitaria%20en%20Ostom%C3%ADas%2FVideo%201%20Revisi%C3%B3n%20del%20material%20para%20el%20cambio%20de%20nuestro%20sistema%2Emp4&referrer=StreamWebApp%2EWeb&referrerScenario=AddressBarCopied%2Eview%2E200e1f4c%2Dbfab%2D457f%2Db9e2%2D8bc28e5a9a67
Video 2 Ostomy system removal	Mendoza Caamaño, Tomás & Pego Pérez, Emilio Rubén	https://nubeusc-my.sharepoint.com/personal/tomas_mendoza_rai_usc_es/_layouts/15/stream.aspx?id=%2Fpersonal%2Ftomas%5Fmendoza%5Frai%5Fusc%5Fes%2FDocuments%2FV%C3%ADdeos%2FEducaci%C3%B3n%20Sanitaria%20en%20Ostom%C3%ADas%2FVideo%202%20Retirada%20del%20sistema%20de%20ostom%C3%ADa%2Emp4&referrer=StreamWebApp%2EWeb&referrerScenario=AddressBarCopied%2Eview%2Ee585f0e7%2D0c34%2D4e70%2Da10e%2D58b57033f018
Video 3 Stoma Cleaning	Mendoza Caamaño, Tomás & Pego Pérez, Emilio Rubén	https://nubeusc-my.sharepoint.com/personal/tomas_mendoza_rai_usc_es/_layouts/15/stream.aspx?id=%2Fpersonal%2Ftomas%5Fmendoza%5Frai%5Fusc%5Fes%2FDocuments%2FV%C3%ADdeos%2FEducaci%C3%B3n%20Sanitaria%20en%20Ostom%C3%ADas%2FVideo%203%20Limpieza%20del%20estoma%2Emp4&referrer=StreamWebApp%2EWeb&referrerScenario=AddressBarCopied%2Eview%2E27681821%2D2e71%2D4a42%2D943d%2D48afbd45b3ad
Video 4 Stoma measurement and plaque trimming	Mendoza Caamaño, Tomás & Pego Pérez, Emilio Rubén	https://nubeusc-my.sharepoint.com/personal/tomas_mendoza_rai_usc_es/_layouts/15/stream.aspx?id=%2Fpersonal%2Ftomas%5Fmendoza%5Frai%5Fusc%5Fes%2FDocuments%2FV%C3%ADdeos%2FEducaci%C3%B3n%20Sanitaria%20en%20Ostom%C3%ADas%2FVideo%204%20Medici%C3%B3n%20del%20estoma%20y%20recorte%20de%20la%20placa%2Emp4&referrer=StreamWebApp%2EWeb&referrerScenario=AddressBarCopied%2Eview%2Ecd7d79dc%2D3edf%2D499c%2Db3f1%2Daa9c8f02409a
Video 5 Installation of 1-piece systems	Mendoza Caamaño, Tomás & Pego Pérez, Emilio Rubén	https://nubeusc-my.sharepoint.com/personal/tomas_mendoza_rai_usc_es/_layouts/15/stream.aspx?id=%2Fpersonal%2Ftomas%5Fmendoza%5Frai%5Fusc%5Fes%2FDocuments%2FV%C3%ADdeos%2FEducaci%C3%B3n%20Sanitaria%20en%20Ostom%C3%ADas%2FVideo%205%20Colocaci%C3%B3n%20de%20sistemas%20de%201%20pieza%2Emp4&referrer=StreamWebApp%2EWeb&referrerScenario=AddressBarCopied%2Eview%2E937555a0%2D0eb5%2D4636%2Da09e%2Dbc6a6a4ccd1f
Video 6 Installation of 2 or 3 piece systems	Mendoza Caamaño, Tomás & Pego Pérez, Emilio Rubén	https://nubeusc-my.sharepoint.com/personal/tomas_mendoza_rai_usc_es/_layouts/15/stream.aspx?id=%2Fpersonal%2Ftomas%5Fmendoza%5Frai%5Fusc%5Fes%2FDocuments%2FV%C3%ADdeos%2FEducaci%C3%B3n%20Sanitaria%20en%20Ostom%C3%ADas%2FVideo%206%20Colocaci%C3%B3n%20de%20sistemas%20de%202%20o%203%20piezas%2Emp4&referrer=StreamWebApp%2EWeb&referrerScenario=AddressBarCopied%2Eview%2Ed2c787af%2D3fb9%2D49f5%2D860a%2D9f04510f66d6
Video 7 Emptying open bags	Mendoza Caamaño, Tomás &	https://nubeusc-my.sharepoint.com/personal/tomas_mendoza_rai_usc_es/_layouts/15/stream.aspx?id=%2Fpersonal%2Ftomas%5Fmendoza

	Pego Pérez, Emilio Rubén	%5Frai%5Fusc%5Fes%2FDocuments%2FV%C3%ADdeos%2FEducaci%C3%B3n%20Sanitaria%20en%20Ostom%C3%ADas%2FVideo%207%20Vaciado%20de%20bolsas%20abiertas%2Emp4&referrer=StreamWebApp%2EWeb&referrerScenario=AddressBarCopied%2Eview%2Ee2532827%2D4b64%2D452f%2D9205%2D3a32db0d73ea

Video S1. Health Education in Ostomies Videos.
