# Supplementary figures and images for: Enhancing Quality of Life in Ostomized Patients Through Smart-Glasses-Supported Health Education: A Pre-Post Study
Source: Healthcare (Basel). 2026 Jan 15;14(2):216. doi: 10.3390/healthcare14020216 (PMC12840625; doi:10.3390/healthcare14020216)

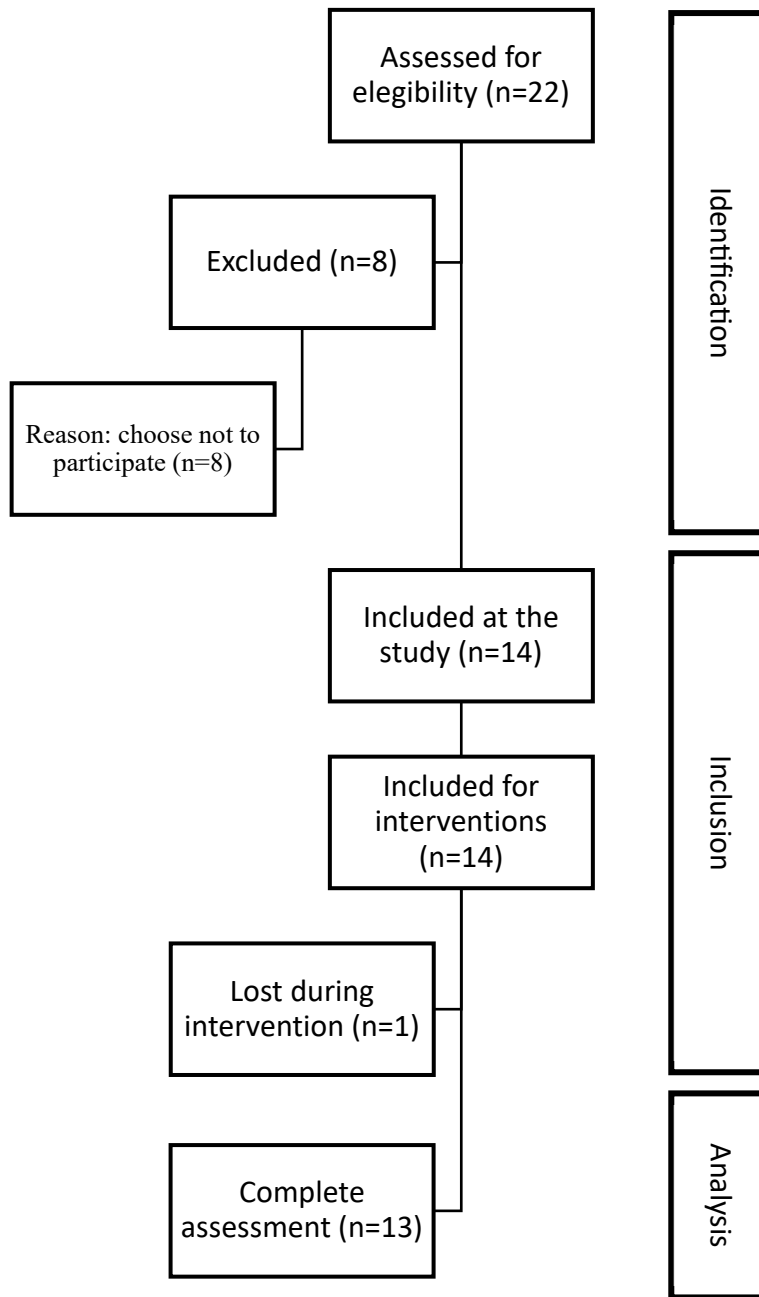

**Figure S1. STROBE Flow chart for observational studies.**

Supplement: Supplementary file 1 [file healthcare-14-00216-s001.zip › Figure S1. STROBE Flow chart for observational studies.pdf]
